# Cardiac patients show high interest in technology enabled cardiovascular rehabilitation

**DOI:** 10.1186/s12911-016-0329-9

**Published:** 2016-07-19

**Authors:** Roselien Buys, Jomme Claes, Deirdre Walsh, Nils Cornelis, Kieran Moran, Werner Budts, Catherine Woods, Véronique A. Cornelissen

**Affiliations:** Department of Rehabilitation Sciences, University of Leuven, Herestraat 49, 3000 Leuven, Belgium; Department of Cardiovascular Sciences, University of Leuven, Herestraat 49, 3000 Leuven, Belgium; School of Health and Human Performance, Dublin City University, Dublin 9, Ireland; Insight Centre for Data Analytics, Dublin City University, Dublin 9, Ireland; Internal Medicine, Division of Cardiology, University Hospital Leuven, Herestraat 49, 3000 Leuven, Belgium

**Keywords:** Cardiac rehabilitation, Technology, Exercise, Physical activity, Lifestyle risk reduction

## Abstract

**Background:**

Cardiac rehabilitation (CR) can slow or reverse the progression of cardiovascular disease (CVD). However, uptake of community-based CR is very low. E-cardiology, e-health and technology solutions for physical activity uptake and monitoring have evolved rapidly and have potential in CVD management. However, it is unclear what the current technology usage is of CVD patients, and their needs and interests for technology enabled CR.

**Methods:**

A technology usage questionnaire was developed and completed by patients from a supervised ambulatory CR program and an adult congenital heart disease clinic and from two community-based CR programs. Results were described and related with age, gender and educational level by Spearman correlations.

**Results:**

Of 310 patients, 298 patients (77 % male; mean age 61,7 ± 14,5 years) completed at least 25 questions of the survey and were included in the analysis (completion rate 96 %). Most (97 %) patients had a mobile phone and used the internet (91 %). Heart rate monitors were used by 35 % and 68 % reported to find heart rate monitoring important when exercising at home. Physical activity monitoring was reported by 12 % of the respondents. Respondents were interested in CR support through internet (77 %) and mobile phone (68 %). Many patients reported interest in game-based CR (67 %) and virtual rehabilitation (58 %). At least medium interest in technology enabled CR was reported by 75 % of the patients. Interest decreased with increasing age (*r* = −0.16; *p* = 0.005).

**Conclusions:**

CVD patients show interest for technology enabled home-based CR. Our results could guide the design of a technology-based, virtual CR intervention.

**Electronic supplementary material:**

The online version of this article (doi:10.1186/s12911-016-0329-9) contains supplementary material, which is available to authorized users.

## Background

Cardiovascular diseases (CVD) are a leading cause of premature death and disability worldwide. An estimated 17.3 million people died from CVD in 2008, representing more than one in four of all global deaths [1]. CVD accounts for over 1.9 million deaths in the European Union annually, making it the single most common cause of death [2]. There is a clear socio-economic gradient, with those in the most deprived areas having consistently higher age-specific prevalence of CVD [3].

Exercise-based cardiac rehabilitation (CR), a secondary prevention programme involving risk factor education, psychological support, medication and exercise has been shown to slow or reverse the progression of CVD and is cost effective [4]. CR is organised in three phases. The first phase of CR relates to the period of hospitalisation following an acute cardiac event and consists of early mobilisation and education. Phase 2 CR consists of supervised ambulatory CR with out-patients required to attend a CR unit two to three times weekly for structured exercise and other lifestyle interventions. The long-term maintenance or phase 3 CR applies when patients leave the hospital-based CR programme and continue exercise and other lifestyle modifications indefinitely. This can be through a community-based exercise programme in a local leisure centre.

Even though CR improves mortality and morbidity rates, uptake of CR, remains suboptimal. Indeed, low levels of participation in phase 2 or 3 CR programs have been reported internationally (14–43 % after myocardial infarction) with high levels of dropout after enrolment [3, 5, 6].

Patient-oriented, medical and healthcare system factors associated with suboptimal participation include availability, affordability and accessibility of a programme, as well as work/domestic commitments and psychological barriers [7]. Current CR delivery approaches do not suit everyone and new innovative ways are needed to match patient preferences in order to improve uptake and compliance to a life-long physically active lifestyle among cardiac patients [8].

E-cardiology and e-health have evolved rapidly and have particular potential in cardiovascular disease prevention and management [9]. Technology solutions for physical activity uptake and monitoring are becoming more readily available and may impact on the current delivery of CR. Some first evidence shows that telerehabilitation solutions for cardiac patients appear to be feasible and effective compared to conventional centre-based CR [10]. However, many different e-health solutions have been employed so far and more research and development is needed. As pointed out by Saner [9] there are still major obstacles to the integration of e-Health and telemedicine into daily clinical practice. One of these main obstacles is the fact that the development of e-Health and telemedicine is still primarily driven technically and not by the needs and expectations of the patients for which the technology is aimed at.

Therefore, the necessity for a study that provides a comprehensive view on the current use of all sorts of technology and the needs and interests in technology enabled CR among patients with various heart diseases was identified. In 2014, Dale at al. [11]. evaluated mobile phone usage and needs and wants from CVD patients for mHealth CR support. They found that mobile phone use was high amongst the participants and that there was interest for receiving CR via mobile technology. However, this pilot study only focused on mHealth and further exploration of other technology solutions for CR is needed. This survey was undertaken as a first step in the process of development, obtaining information of possible useful features from a large group of participants that is needed for the development of the PATHway platform, a European Horizon 2020 funded project (www.pathway2health.eu). This future platform will be designed as an internet enabled sensor-based home exercise platform that will allow remote participation in CR exercise programs at any time and aims to engage patients in a lifelong physically active lifestyle.

## Methods

### Study design and patients

A cross-sectional multicentre study was undertaken in two settings; Dublin City University, Ireland and University of Leuven, Belgium. The protocol for the study received ethical approval from the Biomedical Ethical Committee of the University and University Hospitals of Leuven and from the Dublin City University Research Ethics Committee. All patients gave informed consent.

Study patients were recruited from a supervised phase 2 ambulatory CR program (UZ Leuven, Belgium), two community based phase 3 CR programs (Harpa Leuven vzw, Belgium and MedEx HeartSmart, Ireland) and the adult congenital heart disease clinic (ACHD, Belgium) between April and June 2015.

The questionnaire (Additional file 1) was administered as an online survey (esurv.org) (ACHD and Harpa Leuven vzw members) or by paper and pencil (ambulatory CR Leuven and MedEx HeartSmart).

### Questionnaire

The content and format of the technology usage questionnaire (TUQ) was adapted from a questionnaire which was used in a previous study investigating the role of technology and mHealth in a CVD population [11].

The first version of the TUQ was firstly shared among the collaborators of the PATHway project to facilitate both clinical and technical feedback on content, format and any further information deemed relevant within the development process. Moreover, the TUQ was circulated to collaborator hospital partners and all clinical team members from both study sites. This was an iterative process, which involved five drafts of the TUQ with the multi-disciplinary feedback being incorporated throughout the TUQ development process.

The final prototype was tested with five HeartSmart attendees to assess clarity, readability and ease of use. The same testing was done with the Dutch version of the TUQ. Following some final refinements based on the pilot patients’ feedback (e.g., addition of well-known apps as examples i.e., Skype), the TUQ was finalized (see Additional file 1 for survey questions for CR patients). The survey first presented some brief background information about the study, the study locations and the investigators. Patients were subsequently asked to fill in the questionnaire. The first eight questions of the TUQ were related to basic demographic, clinical and cardiovascular risk characteristics of the cardiac patients. Nine questions were related to current technology usage (mobile phone, internet, computer games, heart rate monitor, and physical activity monitor) of the patients. The last 13 questions were dedicated to the patients’ interests, needs and wants from a technology-based, virtual CR intervention. One of these questions consisted of a table with CR core components, such as exercise, smoking cessation, stress management, etc., where the participant was asked to indicate how useful advice on these components would be for him/her (scores ranging from 0, not at all useful to 5, very useful).

One question of the TUQ was than adapted for use in patients with congenital heart disease. In this TUQ version, the background question ‘for which reason(s) do you attend CR’ was replaced by ‘which congenital heart defect(s) do you have’.

After completing the questionnaire, patients submitted their answers online or returned the hard-copy questionnaires which were then exported to or entered manually in the database for analysis.

### Analysis

Questionnaires were excluded from the analysis when five or more answers were missing. SAS statistical software 9.3 was used to conduct data analyses. Analysis of survey responses was largely descriptive. Items were re-coded such that 1 equalled a positive response (i.e., “yes”), while 0 indicated a negative response. Gender was coded as one for male and two for female. In order to evaluate interest in technology enabled CR, four items were used that assessed interest in technology. The scores on the items indicating interest were then summed, with participant scores ranging from 0 to 4 for overall interest in PATHway. Responses and scores were related to age, gender and educational level by means of the Spearman rank correlation coefficient. Analysis of variance and Chi square tests were used to investigate differences between groups. Statistical significance was set at *p* < 0.05.

## Results

### Patients

Of 310 responders, 298 patients [77 % male, mean age 61.7 ± 14.5 (range 17–83) years] answered at least 25 questions of the survey and were included in the analysis (completion rate of 96 %). The general characteristics of the respondents are summarized in Table 1. The female participants (mean age 51.3 ± 18.6 years) were significantly younger (*p* < 0.001) than the male (mean age 64.8 ± 18.1 years) participants, which could be attributed to a significantly higher number of female participants among the ACHD group (*p* < 0.001). The ACHD group was significantly younger compared to the other study groups (*p* < 0.001). There were no differences regarding demographic characteristics between community based CR patients in Leuven and Dublin (*p* > 0.05 for all).

**Table 1:** Demographic Characteristics of the respondents

	all	Phase 2 CR	Phase 3 CR	ACHD
number	298	56	199	43
age (years)	61.7 ± 14.5	62.5 ± 10.7	67.1 ± 8.12	35.8 ± 14.2
male gender (%)	77	82	82	44
smoking (%)	3	5	3	5
drinking (%)	34	30	38	23

*CR* cardiac rehabilitation, *ACHD* adult congenital heart disease; drinking is defined as consumption of more than 7 units per week. Data are reported as number, as mean ± standard deviation or as percentage

Patients reported at least one of the following cardiac diseases or interventions: Myocardial infarction (11 %), percutaneous coronary intervention (32 %), coronary artery bypass graft (19 %), valve surgery (11 %), pacemaker/implantable cardioverter defibrillator implantation (6 %), heart failure (8 %), ACHD (13 %). Nineteen percent (=56/298 patients) of the patients were participating in a phase 2 CR program; 68 % (=199/298) in a phase 3 CR program, 43 of the remaining patients (13 %) were ACHD patients who were not formally participating in a CR program.

Most of the patients exercised 3 times per week (41 %), 38 % of the patients exercised 4 or more times/week and the remaining patients exercised twice or less times per week.

Overall, patients had a high educational background. Twenty three percent (70/298) of the respondents reached at least a Master’s degree, 36 % of respondents reported to have obtained a Bachelor’s degree, 24 % of the respondents attained only a secondary school level and the remaining 17 % of the patients reached a primary grade or secondary lower grade. It has to be noted that 5/43 of the participants with congenital heart disease were university college or university students. With regard to the community based CR participants (phase 3), Harpa participants (Belgium) had a significantly higher level of education when compared to the HeartSmart (Dublin) cohort (*p* = 0.0018).

### Current technology usage

Of all the patients surveyed, 91 % regularly accessed the internet; 76 % of them used the internet every day. Most of the internet users access the internet via a personal computer (95 %), tablet (44 %) and/or smartphone (43 %).

Almost all patients owned a mobile phone (97 %); in 64 % of them it was a smartphone. Smartphone users were younger than mobile phone users (*r* = 0.42; *p* < 0.001), with no difference between men and women. Main application areas on the phones were calls (70 %) and sending text messages (66 %). Twenty five percent of all patients (75/185 or 41 % of smart phone users) reported to use Apps on the smartphone. Use of a smartphone for Apps, instant messaging (56/185, 30 %), social networks (50/185, 27 %) and gaming (23/185, 12 %) was more frequent in the younger patients (correlations with age of respectively *r* = −0.35, *r* = −0.33, *r* = −0.43 and *r* = −0.36, respectively; *p* < 0.001). Smartphones were used for internet searching, Apps, instant messaging and social networks more often by females when compared to males (*p* = 0.003, *p* = 0.28, *p* = 0.09, *p* < 0.001 and *p* < 0.001, respectively). However, when both age and gender were correlated to various smartphone usage utilities, only age remained a significant determinant (*p* < 0.001).

Seventy eight percent of patients (222/283) stated that they had never used a computer based physical activity game, whereas 69 % (202/292) of the patients reported to know what these types of games are or to have seen it before. Younger patients more often reported to have used computer based physical activity games (*r* = −0.32; *p* < 0.001). In our cohort, the knowledge and use of physical activity games was not different between genders.

Heart rate monitors to measure heart rate during exercise sessions were used by 35 % of all patients, without any difference between phase 2 CR, phase 3 CR and ACHD patients (*p* > 0.05). Over two thirds of patients (68 %) that were not using a heart rate monitor, perceived heart rate monitoring to be important during home exercises. Differences between the study settings were present. Within the phase 3 CR group, the use of a heart rate monitor was significantly (*p* < 0.001) higher in patients participating in Belgium (51 %) compared to Ireland (6 %). On the other hand, significantly more HeartSmart attendees reported heart rate monitoring to be important for home-based exercise when compared to Harpa attendees (66 % and 35 % respectively). The use of a heart rate monitor was not related to educational background. A small though significant relationship with age was present (*r* = −0.15, *p* = 0.012).

Twelve percent of the patients reported to use some sort of physical activity monitoring (steps, energy expenditure) by means of currently available devices or smartphone Apps on the market. The use of these devices was not related to age or educational level and no differences between groups were found.

### Patients’ interests and preferences for technology enabled CR

When asked whether patients would be interested in receiving CR support through the internet, 77 % responded positively. These patients would like to receive this support through e-mails (65 %), websites (39 %) and online video’s (36 %). Interest in internet based CR support was higher in patients with a higher educational level (*r* = 0.14; *p* = 0.014) but was not related to age or gender, and was not different between groups/centres.

Sixty-nine percent of the patients surveyed reported to be interested in receiving CR support via mobile phone. Text messages were most popular (46 %), followed by Apps (30 %). Interest in CR support through a mobile phone increased with decreasing age (*r* = −0.15; *p* < 0.01) and was higher in the younger ACHD group compared to the others (*p* = 0.002). In the ACHD group, apps were more popular than text messages.

**Fig. 1:**
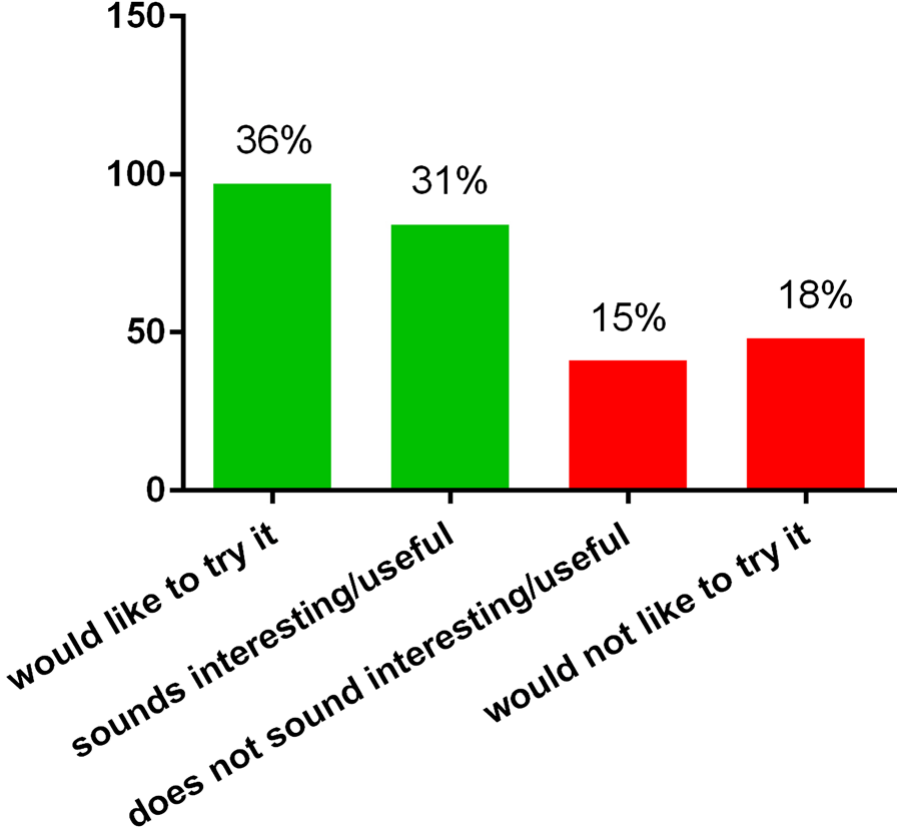
Answers to the question “How would you rate a home rehabilitation exercise program through a computer game?”

**Fig. 2:**
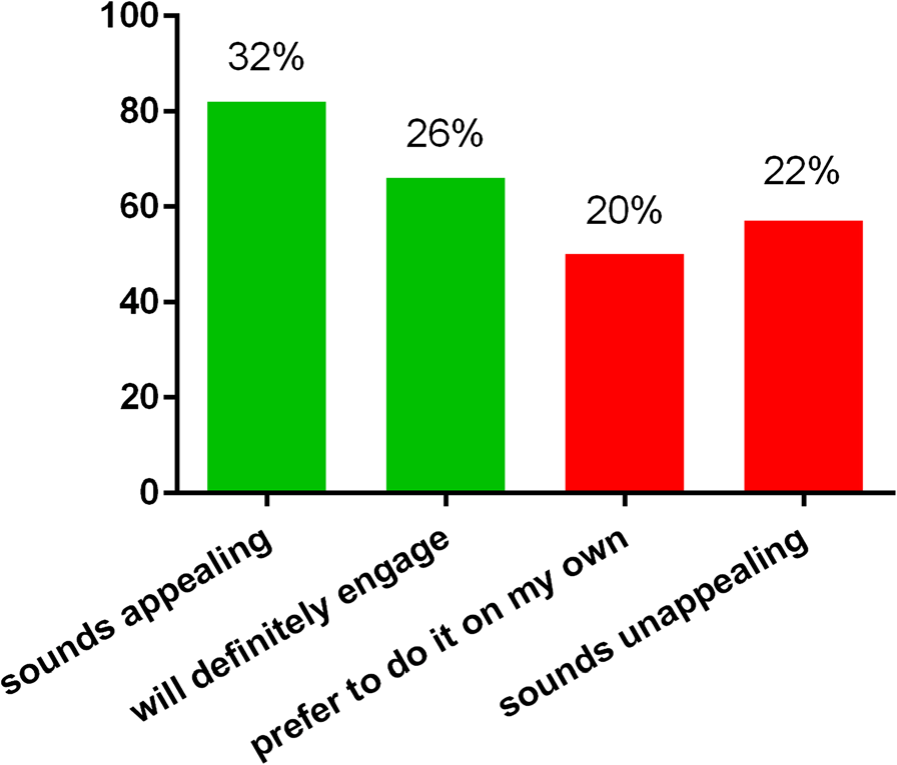
Answers to question “Would you think that a virtual rehabilitation class would be useful?

Although 86 % of the patients were already enrolled in a supervised training program, 67 and 58 % of the patients reported to be interested in a home rehabilitation exercise program through a computer game or exerclass with a virtual coach, respectively (Figs. 1 and 2). The question regarding patient preferences for the interaction with such an online platform revealed that 38 % of the patients prefer an interaction of no more than a few mouse clicks, another 38 % do not mind about using more than a few mouse clicks and 24 % of patients would prefer access to a full menu of interactions to be in control of the game.

**Fig. 3:**
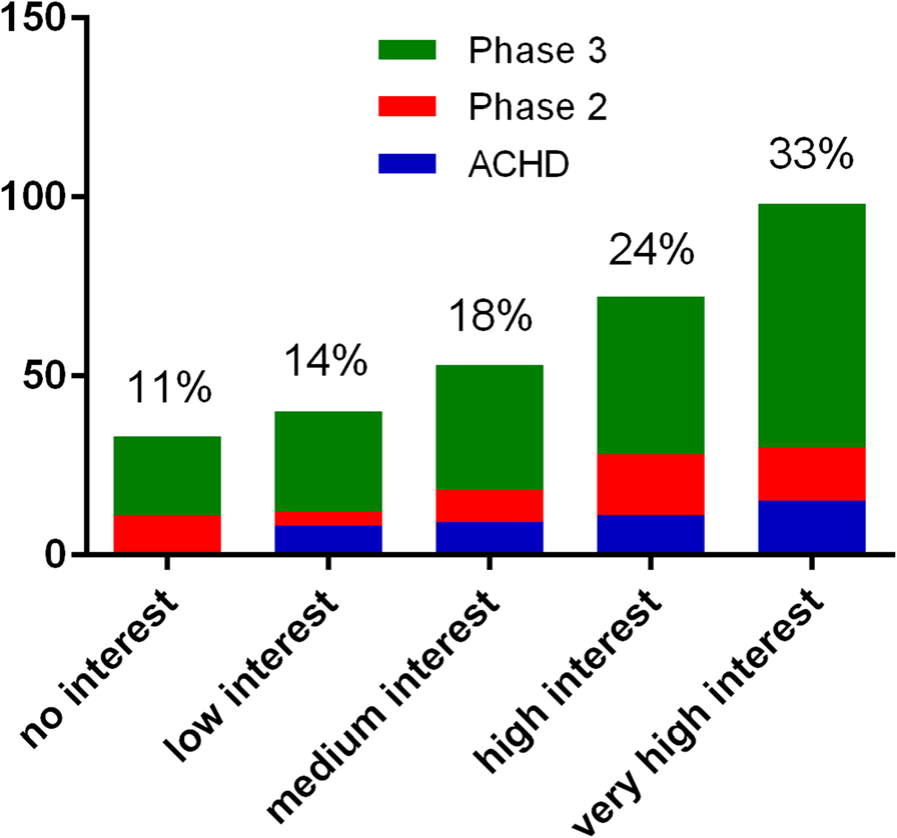
Overall interest in technology enabled CR

**Fig. 4:**
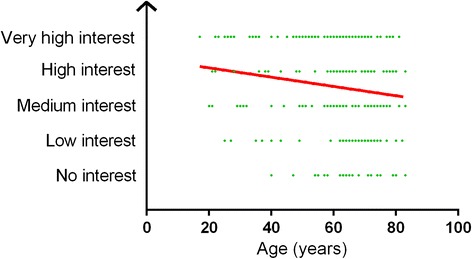
Interest in technology enabled CR in relation with age

Overall interest in online home-based exercise platforms with remote monitoring and feedback, like the future PATHway platform, is shown in Fig. 3. Seventy five percent of the respondents reported at least a medium interest in technology enabled CR. Overall interest was similar across groups, decreasing with increasing age (*r* = −0.164; *p* = 0.005; Fig. 4) and independent from gender and educational background.

**Fig. 5:**
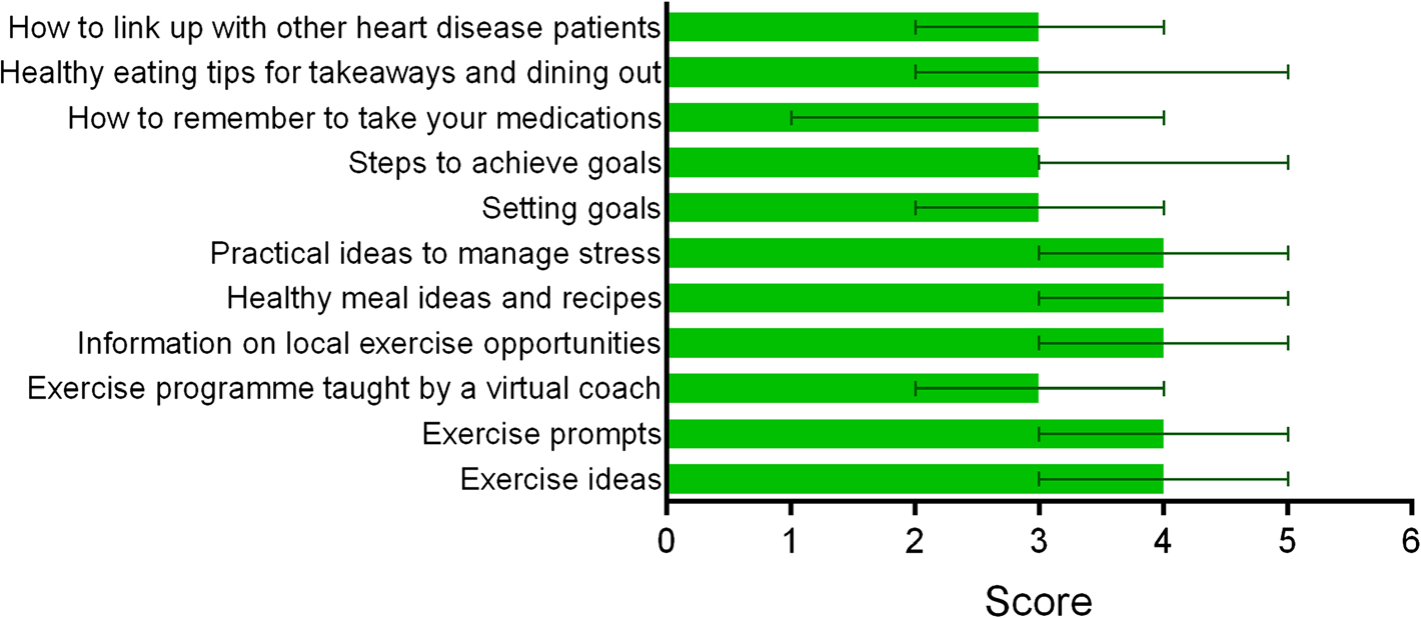
Median and interquartile ranges for scores on the importance of CR components

Median scores and interquartile ranges of the usefulness of advice on separate CR components are listed in Fig. 5. Advice on exercise ideas, exercise prompts, information on local exercise opportunities, healthy meal ideas and recipes and practical ideas to manage stress received the highest ratings for inclusion in a technology based CR platform. Ratings were not related to age, gender and educational background, with the exception of support with medication adherence where participants with a lower educational background were more likely to indicate medication adherence support as very important.

## Discussion

This study documents current technology use of cardiac patients and their interest in technology enabled CR. This research is needed given the increasing use of rapidly evolving technology in health care. Therefore, for the successful development of home-based CR solutions it is important to capture patterns of technology use and patient interests. The main survey results show that patients with cardiac disease, young and old, are regularly using the internet and mobile phones, although the use of active video games is less established. Moreover, cardiac patients show a clear interest in receiving CR support through the internet, and to a lesser extent through their mobile phone. A second finding from our research was that the majority of patients show interest in technology-enabled CR and that this interest was higher in the younger patients indicating great potential for future CVD patients.

### Technology use

The internet use observed here is high. Only Peels et al. [12] reported in 2010 similar numbers in a healthy Dutch cohort with ages between 55 and 65 years, however, in their age group of over 65 years, this percentage decreased to 68 %. Other studies, performed in the same time period, have shown that, 67 and 77 % of men and women aged 50–64 years were using the internet in the United States of America, and also 43 % of Slovenians older than 65 years [13–15]. Age is considered a major determining factor when it comes to technology use. However, our as well as other results show that the internet can be a useful tool to support cardiac patients of all ages and that age currently is certainly not to be seen as an immovable barrier for web-based interventions [12, 16].

Concerning mobile phone use, with 97 % mobile phone and 64 % smartphone ownership, our study is in line with the findings from Dale et al. [11] who also studied CR patients. Similarly, in the older population in general, mobile phones and smartphones are used by the vast majority of men and women [13, 14, 17]. Despite the widespread use of smartphones in our population, current interest in a smartphone App for CR support was lower compared to interest in internet based CR support. However, interest was significantly higher in the younger population compared to the older population which keeps the opportunities for future SMS and App use open. Our findings are in line with Pouchieu et al. [18] who investigated a French, mostly female (76 %) population with a mean age of 51.2 years, demonstrating low interest in Apps, but also observed that younger persons showing higher interest. Further qualitative research is necessary to complement our findings and guide a CR intervention with regards to choices on mobile phone and/or internet use.

The low rates (22 %) of game-experience seen in our study population are comparable to the results of Scanlon et al. [17], who reported that only 3 % of their investigated cohort used a game console. This option for delivering CR is thus certainly subject for further research.

Heart rate monitors were used by one third of the studied cardiac population, despite the fact that almost all of them exercise in a supervised setting. Moreover, 68 % of the patients that did not use a heart rate monitor reported that they find heart rate monitoring important for home based exercise. This might mean that patients who exercise at home will be more likely to use a heart rate monitor. Since many years, heart rate zones based on maximal symptom limited exercise testing constitute the golden standard for defining the exercise training intensity for patients with heart disease [19–25]. Therefore, it can be assumed that the respondents on this TUQ are aware of how to use heart rate zones when exercising on their own, however evaluation of heart rate monitor use amongst patients not engaged in supervised CR is necessary to complement our findings. Given the reported interest in heart rate monitoring during home exercise, the importance of heart rate based training, along with the large availability of heart rate monitors on the market, this is a feature a technology enabled CR platform should implement.

Physical activity monitoring is a very modern lifestyle trend and new Apps and devices are continuously being introduced. Nevertheless this type of monitoring is only scarcely adopted by the patients in our study. So far, no randomized controlled study was able to establish the effectiveness of physical activity monitoring for increasing physical fitness and decreasing mortality of patients with cardiac disease. Moreover, many questions with regard to validity and reliability of these devices remain. In that light, physical activity monitors do not seem useful for monitoring of CR but rather have their potential for increasing the motivation to live a healthy and active life.

### Relation between technology use and age, gender and educational level

The main reasons for the somewhat lower use of technology in older patients to date has been suggested to be related to cost, privacy concerns and the fear that technology may be impractical to use and too noticeable within their homes [26, 27]. Generally, other cited factors influencing the acceptance of technology in older adults are ‘ease of use’, ‘usefulness’ and ‘self-efficacy’ [14, 16, 28]. Our findings confirm this in part, given that 38 % of the patients prefer an interaction of no more than a few mouse clicks. Nevertheless, a quarter of the current cohort reported to prefer a full menu of possibilities enabling them to be in control of the game or program. This suggests a simple interaction method may be preferable, but with the possibility to apply advanced settings if the patient is interested. However, this wide distribution of preferences warrants further investigation and testing by end-users during development stages in order to tailor a technology-based CR intervention to the needs of todays’ patients.

In line with previous research [28, 29], we observed no gender difference with regard to internet use, smartphone ownership, gaming or interest in internet delivered support for CR [28, 30]. However, literature shows that men and women might be using current available technology differently. Men tend to use the internet in a broader way, including more frequent playing of games [30, 31]. In a study conducted in young and adolescent people, boys reported higher levels of enjoyment when playing competitive exergames, while girls were more likely to enjoy games with a high cooperation level [32]. Pouchieu et al. [18] reported that women seem to have lower computer skills in comparison with men. However, women reported a more frequent use of the worldwide web to search for health information [33]. It seems that in order to reach the entire target population of CVD patients, a future technology enabled CR program should consist of a variety of technology solutions to reach both men and women.

Blazun et al. [15] noticed a difference in mobile phone use with regard to educational level, with higher level of education leading to more utilization [15] and more frequent use of different functions on mobile phones [30]. In this regard, the relatively high technology use of our cohort in comparison to what is reported for similar age groups might be partly attributable to a higher educational background. The cohort we studied here reported a high educational background, which is however representative to the Belgian population of similar age-range (17–83 years) [34]. Our high engagement with technology might therefore also partly be driven by high levels of education in Belgium. This is confirmed by a recent review of health care technology usage where 68 % of studies concluded that higher education resulted in greater acceptance of technology use [28]. Also Kontos et al. [33] mention socio-economic status (education, income or both), age and gender as the most significant influencing factors with regard to eHealth usage.

### Overall interest in technology enabled CR

CR is a multidisciplinary program with exercise training as the core component, which is complemented with education and support regarding a healthy diet, stress management and cardiovascular disease management. In this study, the participants were asked which CR components they rate as most important to be included in a technology enabled CR platform. The results point towards most interest in exercise related support, namely exercise ideas, exercise prompts and local exercise opportunities. Furthermore, participants also reported that support about healthy meal ideas and practical ideas to manage stress were very important.

With 75 % of the participants reporting at least a medium interest in technology enabled CR, we can conclude that there is a strong rationale for the development of technology enabled CR programs; especially since this number will probably grow over the years. The young technology users of today constitute the basis of tomorrow’s CVD population leading to a technology ‘native’ target group of older adults, who will be highly engaged with technology. Also worth noting is that two third of the patients with ACHD report to be interested in technology based CR. This group currently is not systematically offered CR, but it is known that they have many questions with regard to exercise and sports participation [35]. The reported interest for technology enabled CR was not related to any of the investigated sociodemographic characteristics, nor to current technology literacy or to type of heart problem.

The current study can further inform the development of a technological approach to CR. A home based technology enabled CR program should be designed such that it is attractive to patients of all ages and backgrounds and as much as possible to all levels of technological literacy. When designing the interface and content of the PATHway platform, the aim should be a system that is ‘easy to use’, ‘useful’ and ‘adapted to the physiological characteristics of the patient’. Apart from the exercise core component in the CR system, attention should be given towards other components, including practical support with healthy meals and stress management. However, our findings should be complemented with further, more in-depth investigation of technology literacy, needs and wants of CVD patients with regards to a technology enabled home-based CR platform.

### Limitations

Our study has some limitations. First, our recruitment strategy did not enable us to define how many potential patients were approached for the study and as a consequence, the response rate could not be calculated. Second, it might not be appropriate to generalize the results of this two-centre study because convenience sampling was used for gathering the data and sampling bias might exist. Third, we reported on a highly active group of cardiac patients and observed a high educational level which might have favoured our participants to more interest in home-based CR and better engagement with technology. Fourth, the questionnaire design we used for this study was intended to reach a broad audience in a feasible way, however this design is less suitable for obtaining more in-depth information with regard to the needs and expectations of the target group. Our results should therefore be complemented with information based on qualitative research.

## Conclusions

This study documents a high usage rate of technology and high level of interest in technology enabled home-based CR which can guide the design of technology-based, virtual CR interventions. While questions remain (e.g. how effective are technology driven exercise training programs? How may/should prompts be utilized? What type of information do patients prefer?), the use of CR programs involving e-health technology seem promising for both men and women with different educational backgrounds. In addition, the likelihood of uptake may be even higher in the future due to the higher use of technology in our younger cardiac patients”.

## Abbreviations

ACHD, adult congenital heart disease; CR, cardiac rehabilitation; CVD, cardiovascular disease; TUQ, technology usage questionnaire

## Additional files

Additional file 1: Technology user questionnaire. (PDF 144 kb)
