# Supplementary material for: Cardiac patients show high interest in technology enabled cardiovascular rehabilitation
Source: BMC Med Inform Decis Mak. 2016 Jul 19;16:95. doi: 10.1186/s12911-016-0329-9 (PMC4949752; doi:10.1186/s12911-016-0329-9)
Supplement: Additional file 1: — Technology user questionnaire. (PDF 144 kb) [file 12911_2016_329_MOESM1_ESM.pdf]

Technology Usage Questionnaire

Research Participation Number
(will be given by researcher)

Your research participation number means that this questionnaire will be <u>anonymous</u>-instead of your name you now have a number <u>just for you</u>.

The <u>consent form</u> with your name will be separated from this questionnaire.

They will only be matched back together if you wish to withdraw from the study.

Technology Usage Questionnaire

Please tick (✓) ONE box only.

Q1: Are you male or female? O Male **O** Female

Q2: How old are you? write in e.g., 63) _______________ years old

Q3: What is your current employment status:

O Employed for wages
O Self-employed
O Out of work and looking for work
O Out of work but not currently looking for work
O A homemaker
O A student
O Retired
O Unable to work

Q4: What is the highest level of education you have completed?

O Some primary (not complete)

O Primary or equivalent

O Intermediate/junior/group certificate or equivalent

O Leaving certificate or equivalent

O Diploma/certificate

O Primary degree

O Postgraduate/higher degree

O None

Q5: How often do you attend exercise classes:

O less than 1 time/week

O 1 time/ week

O 2 times/week

O 3 times/week

O more than 3 times/week

Q6: Do you smoke? O Yes……. O No……

Q7 Do you drink alcohol regularly (i.e. more than 7 drinks/week)?

O Yes O No

Q. 8 For which reason(s) do you attend cardiac rehabilitation? (Tick (✓) all that apply)

O heart attack
O stenting
O bypass grafting
O valve surgery
O pacemaker/ICD
O heart failure

Q9: Do you have a mobile phone or smartphone?

Please tick (✓) ONE box only

O Yes…… (go to question 10) O No……. (go to question 18)

Q10. What functions do you use on your mobile phone/ smartphone? (Tick (✓) **all that apply)**

O Phone calls
O Text messaging
O Camera
O Receive videos and/or photos
O Internet search (e.g., google)
O Applications ('apps' e.g., skype, google maps, hailo)
O IM (instant messaging, e.g., viber, whatsapp etc.)
O Social networks (e.g., facebook)
O Gaming applications (e.g., angry birds, candycrush)

<u>Q11:</u> Would you be interested in receiving continuing advice cardiac rehabilitation support via your mobile phone/smartphone?
Please tick (✓) ONE box only

O Yes (go to question 13)   O No (go to question 12)

<u>Q12</u>: If no, why not? Please explain briefly.

____

____

<u>Q13:</u> If yes, which types of mobile phone/smart phone communication would you like? (Tick (✓) all that apply)

O Text message
O Video clip
O Smartphone App
O Internet
O Emails
O Other:
(please list)

____

<u>Q14:</u> If there was a free program that offered these types of communication from your Cardiac Rehabilitation medex program would you sign up?

O Yes……. (go to question 15)
O No……… (go to question 16)

<u>Q15:</u> If yes, how many messages would you like to receive?

O less than 1-2 messages per week
O 1-2 messages per week
O 3-4 messages per week
O 1 message per day
O 2 messages per day

<u>Q16:</u> Do you have regular access to the <u>Internet</u>?

Please tick (✓) ONE box only

O Yes (go to question 17)……… O No (go to question 19)

<u>Q17:</u> If yes, how often do you use the internet?

O everyday
O 3 or more times per week
O once per week
O less than once per week

<u>Q18:</u> What device do you use to access the internet? (Tick(✓) all that apply)

O Personal computer (e.g. desktop/ laptop)
O Tablet (e.g. ipad, ipad mini etc.).
O Phone
O Other (please specify)____________________

<u>Q19:</u> Would you be interested in receiving continuing cardiac rehabilitation support via the internet?

Please tick (✓) ONE box only

O Yes (go to question 21)………………………… O No (go to question 20)

<u>Q20:</u> If no, why not? Please explain briefly:

____

____

<u>Q24:</u> If yes, which types of internet communication would you like? (Tick(✓) all that apply)

O E-mails
O Educational Video
O Web sites
O Information in leaflet form
O Other:
(please list)

____

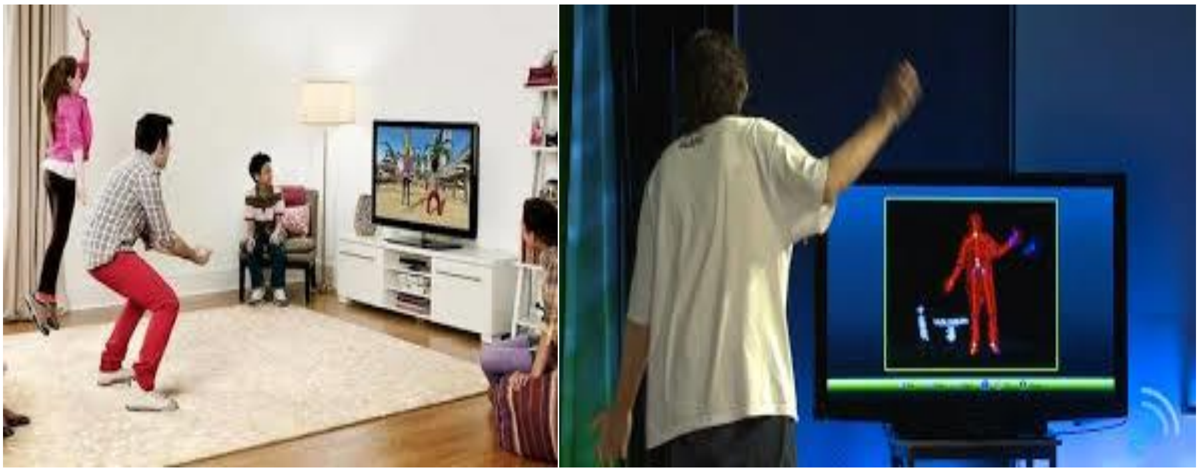

<u>Q21:</u> Are you aware of computer based physical activity games (e.g., Nintendo Wii, Microsoft Kinect (Xbox), such as those in the pictures above)? (perhaps your children own an Xbox)?

O Yes, I know what it is
O Have seen it before
O No

<u>Q22</u>: Have you used it before?
O Yes, I have used it
O No, I have never used it

<u>Q23:</u> How would you rate a home rehabilitation exercise program, similar to a Nintendo Wii/ Kinect Xbox computer game?
O I would like to try it
O Sounds interesting/useful
O Does not sound interesting/useful
O I would <u>not</u> like to try it

<u>Q24:</u> How much interaction with such a game platform would you tolerate?
O Only really few mouse clicks to start exercising
O Would not mind more than a few
O Would prefer to have a full menu of interaction, to be in control of the game

<u>Q25:</u> Would you think that a virtual rehabilitation class (where you could exercise with other patients and virtual coach) would be useful?
O Sounds appealing
O Will definitely engage
O Prefer to do it on my own
O Sounds unappealing

Q26: How useful would the following advice be?

Please tick (✓) a box between 1-5 for each line)

	1 Not at all useful	2	3 Somewhat useful	4	5 Very useful
Exercise ideas					
Exercise prompts					
Exercise programme taught by a virtual coach (via the internet)					
Information on local exercise opportunities					
Healthy meal ideas and recipes					
Practical ideas to manage stress					
Setting goals					
Steps to achieve goals					
How to overcome cigarette cravings					
How to remember to take your medications					
Healthy eating tips for takeaways and dining out					
How to link up with others who are living with heart disease					

Q27: If there is any advice that you would find useful that is not mentioned above please list:

____

____

Q28: **Do you currently use a heart rate monitor while exercising?**

O Yes .... O No (go to question 30)

Q29: **If no, would you like your heart rate to be monitored by a wrist watch device when you exercise at home?**

O Yes .... O No

Q 30: **Do you currently wear any other type of device which records your physical activity levels (e.g. fitbit, pedometer)**

O Yes .... O No

Should you have any other comments, please provide them here:

____

____

____

____

Thank you very much for taking part.
